# Designing for Change: Principles for STEM Programs That Foster Organizational Transformation

**DOI:** 10.1177/15381927261418040

**Published:** 2026-02-18

**Authors:** Edwin Jose Perez

**Affiliations:** 1 The University of Texas at El Paso, USA

**Keywords:** STEM intervention programs, organizational change, STEM equity

## Abstract

This study examines how STEM programs can be intentionally designed to serve as mechanisms for organizational transformation. Grounded in organizational change theory and findings from a multi-site qualitative case study of the Meyerhoff Adaptation Project, the study presents four design principles that illustrate how intentional choices can reconfigure structures, cultures, and power dynamics, which enable inequity within higher education. The principles offer guidance for developing sustainable, equity-centered STEM initiatives that advance institutional change.

## Resumen

Este estudio examina cómo los programas de STEM pueden diseñarse intencionadamente para servir como mecanismos de transformación organizativa. Basándose en la teoría del cambio organizativo y en los resultados de un estudio de caso cualitativo realizado en múltiples centros sobre el Proyecto de Adaptación Meyerhoff, el estudio presenta cuatro principios de diseño que ilustran cómo las decisiones intencionadas pueden reconfigurar las estructuras, las culturas y las dinámicas de poder que permiten la desigualdad en la educación superior. Los principios ofrecen orientación para desarrollar iniciativas STEM sostenibles y centradas en la equidad que promuevan el cambio institucional.

## Background and Statement of the Problem

Addressing the underrepresentation of people from racially minoritized backgrounds (Black, Latinx, Indigenous) in Science, Technology, Engineering, and Math (STEM) fields has remained a persistent challenge for decades (National Academies of Sciences Engineering and Medicine [NASEM], 2023). These enduring inequities have long been documented by leading agencies such as the National Academies of Science, Engineering, and Medicine (NASEM), a congressionally chartered nonprofit organization that provides independent, evidence-based advice to the federal government and helps shape national STEM policy (NASEM, n.d). Recent findings reveal that while Black, Latinx, and Indigenous people represent more than 30% of the employed U.S. population, they account for only 23% of the STEM workforce with bachelor’s degrees or higher (NASEM, 2023). This persistent underrepresentation underscores the need to interrogate the approach that higher education institutions have taken to structure opportunity and access to STEM fields.

In response to persistent disparities, federal agencies and private foundations have invested heavily in institutional efforts to promote greater equity, most notably through the development and implementation of STEM intervention programs (SIPs; George et al., 2019; NASEM, 2016, 2023; Tsui, 2007). SIPs vary in their goals, components, and target populations, yet are unified in their purpose to increase the representation of students historically excluded from STEM, including students of Color, women, low-income, and first-generation students (George et al., 2019). SIPs have similarities with general institutional initiatives, such as mentoring programs, in that they seek to provide supports for students; however, they differ in their explicit focus on addressing the barriers that have long contributed to underrepresentation in STEM (George et al., 2019). Accordingly, they function as strategic initiatives designed to intentionally recruit, retain, and graduate underrepresented students in STEM fields through varying programmatic services and components.

Research on SIP efficacy has demonstrated that participation in these programs can lead to positive impacts in a myriad of academic and non-academic outcomes for students from underrepresented groups, including improvements in GPA, retention, and graduation rates, sense of belonging, science identity development, and greater likelihood of pursuing STEM careers (Abrica et al., 2022; Maton et al., 2012; NASEM, 2016; Pearson et al., 2022; Shortlidge et al., 2024). This documented success has helped solidify SIPs as the dominant institutional strategy for improving STEM outcomes among students from underrepresented groups. Reflecting this consensus, the National Academy of Sciences, National Academy of Engineering, & Institute of Medicine (2011) have asserted that every 4-year institution should develop and implement SIPs based on proven models, in order to expand opportunity and participation in STEM.

However, a growing literature base has questioned whether these programs can truly lead to broad and systemic change. Namely, scholars have critiqued SIPs for concentrating on affecting student-level outcomes while leaving harmful and exclusionary institutional structures and cultures largely intact (Asai, 2020; Linley & George-Jackson, 2013; McGee, 2020; Perez, 2023; Robinson, 2022). This scholarship argues that centering student-level change rather than organizational transformations may inadvertently reinforce the very inequities that they seek to change. While there is growing consensus that we must work toward organizational change, limited research has examined how initiatives, like STEM intervention programs, can be catalysts for broader organizational change (López et al., 2022; Reinholz et al., 2021).

This article advances our understanding of how STEM initiatives can contribute to organizational transformation through intentional program design. The study examines how SIPs can be organizationally structured to foster sustainable, equity-centered change within higher education institutions. As such, the study is guided by the following research question:

1. How can STEM intervention programs (SIPs) be organizationally designed to enable sustainable, equity-centered change within higher education institutions?

Grounded in organizational theory and findings from a multi-site qualitative case study of the Meyerhoff Adaptation Project (MAP), the article presents four design principles that illustrate how SIPs can be organizationally structured to cultivate durable and expansive equity-centered change, thereby providing a framework to guide future institutional efforts across the higher education ecosystem.

## Literature Review

### STEM Intervention Programs in Higher Education

 Systematic reviews of the STEM intervention literature (Palid et al., 2023; Pearson et al., 2022) reveal a consistent pattern: the SIP literature has overwhelmingly focused on documenting programmatic outcomes and identifying common elements employed, while far less attention has been given to organizational processes such as *how* SIPs are implemented, sustained, or working toward enacting broader organizational change. Numerous studies (Carpi et al., 2013; NASEM, 2016; Rincón & George-Jackson, 2016; Shortlidge et al., 2024; Tsui, 2007), along with recent systematic reviews of the SIP literature (Palid et al., 2023; Pearson et al., 2022), have documented the core components commonly employed across these programs. These studies have demonstrated that SIP elements often include: summer bridge programs; academic support services such as tutoring, workshops, and study skills development; mentorship opportunities from both institutional agents and peers; financial assistance; spaces for community building (e.g., living-learning communities, cultural events, and networking activities); and career development supports like career counseling, graduate school preparation, research experiences, and internships. These various components reflect a range of institutional strategies that can be employed to work toward cultivating greater student success in STEM.

 Although numerous interventions are available to institutions, research on SIPs and other student support initiatives has shown that many programs address a single issue (e.g., providing mentoring or financial aid) and operate in isolation from one another (Kezar & Kitchen, 2020). Comprehensive programs, those that offer a broad range of services and supports under one program, remain uncommon in STEM (Kezar & Holcombe, 2020; Kezar & Kitchen, 2020). However, multiple studies have consistently identified the Meyerhoff Scholars Program (MSP) at the University of Maryland Baltimore County (UMBC) as one of the few exemplars of a comprehensive model that has proven to cultivate student success and has worked toward creating broader organizational change (Hrabowski et al., 2019; Kezar, 2019; Kezar & Kitchen, 2020; Palid et al., 2023; Pearson et al., 2022). The Meyerhoff program is thus commonly seen as the exception rather than the norm among SIPs in higher education. Moreover, scholars have suggested that its success is closely tied to the institution’s leadership (Kezar & Holcombe, 2020).

 While research from the Meyerhoff program has demonstrated that SIPs have the potential to advance meaningful change, such examples are very rare. Most SIPs operate under an intervention paradigm that emphasizes improving individual student outcomes rather than transforming the broader environment that shapes those outcomes. The following section examines the common assumptions that underlie SIPs that are guided by an intervention paradigm and its implications for STEM equity.

### Rethinking the Interventions Design Paradigm

Critiques of SIPs have largely focused on the foundational assumptions embedded in the intervention paradigm they employ. SIPs, by design, largely focus on preparing students to navigate and succeed within educational environments that have been shaped by the groups that have historically dominated the STEM field, namely, those who are white, male, and middle to upper class (Calabrese Barton et al., 2018; McGee, 2016, 2020). Extensive research has demonstrated that STEM fields are marked by deeply embedded norms and ideologies centered on individualism, hyper-competition, myths of meritocracy, gender bias, and color evasiveness, which have contributed to the marginalization of women and students from racially minoritized backgrounds (Battey & Leyva, 2016; Calabrese Barton et al., 2018; McGee, 2020; NASEM, 2016, 2018, 2023) These logics create the cultural and structural features of STEM disciplines and enable the maintenance of harmful STEM environments that students from underrepresented backgrounds must endure (McGee, 2020; NASEM, 2023). When SIPs prioritize helping students adapt to these settings without also addressing the broader organizational conditions that produce marginalization, they risk reinforcing the very inequities they were created to dismantle.

 Understanding harmful STEM norms, ideologies, and cultures is important because they are pillars that enable hostile environments to be sustained (NASEM, 2023). Moreover, it illuminates that STEM success is not just a product of individual effort but rather a social process shaped by broader institutional forces (Carlone & Johnson, 2007; Hurtado et al., 2011). Research demonstrates that students of Color in STEM often face racism through racial stereotypes and microaggressions (Beasley & Fischer, 2012; Lee et al., 2020), low expectations from faculty and peers (Hurtado et al., 2011; McCoy et al., 2017), and must continuously prove their intellectual abilities (Leyva, 2021; McGee & Martin, 2011). Importantly, women of color face a “double bind” in STEM where they are excluded based on both their race/ethnicity and gender (Malcom et al., 1976), resulting in facing interlocking barriers shaped by racism and sexism as they navigate STEM spaces (Leyva, 2021).

Collectively, these findings challenge the assumption that equity in STEM can be achieved through student-focused solutions alone. If exclusion is embedded in the structures, norms, and routines of STEM disciplines themselves, then any effort to create meaningful change must directly challenge these systemic issues (Asai, 2020; Linley & George-Jackson, 2013; McGee, 2020; Perez, 2023; Robinson, 2022). Framing success as an individual act of resilience defines achievement on the basis of one’s ability to endure harm, leaving institutions unchallenged and unchanged. As Robinson (2022) powerfully argues, “the responsibility for reform must shift from fixing students to fixing institutions” (p. 80). A growing body of research echoes this call, urging a move away from student-level outcomes, which often reinforce deficit-based logics, toward approaches that identify and dismantle the institutional conditions that perpetuate inequity (Asai, 2020; Castro, 2014; Linley & George-Jackson, 2013; McGee, 2020; NASEM, 2023; Perez, 2023; Robinson, 2022). Achieving this goal of transformative STEM reform will require a fundamental shift in perspective, one that understands that organizations should be the primary targets of change, not students.

### Bridging the Gap: Integrating Organizational Perspectives to SIP Design

Although the imperative to shift the burden of reform from students to institutions is gaining traction, most STEM intervention programs remain unequipped to catalyze institutional transformation. One of the primary reasons is that STEM education and organizational change theory have evolved in silos, rarely informing each other in substantive ways (Henderson et al., 2011; Laursen, 2019; López et al., 2022; Reinholz et al., 2021). This disconnect helps explain why existing STEM initiatives have largely focused on individual student outcomes, while overlooking the organizational conditions that perpetuate inequity.

 This disciplinary divide is well documented in the literature. For example, a systematic review by Reinholz et al. (2021) analyzed how theories of change have been applied to STEM reform efforts between 1995 and 2019 and found that the vast majority of studies focus on individual actors such as faculty and students, while organizational change theory is used only superficially. A key insight raised by Reinholz et al. (2021) is that many of the individuals (principal investigators, faculty, etc.) involved in enacting and leading STEM initiatives often come from traditional STEM backgrounds (e.g., biology, computer science, engineering) and thus their training and knowledge base is rooted in the epistemologies and norms of the sciences rather than the social sciences, where organizational theory has been more robustly developed. While these individuals may be deeply committed to advancing broader change, they lack formal training in theories of change and often have limited opportunities to engage in what Reinholz et al. (2021) call “change education” (p. 19). These disciplinary silos help explain why many well-intentioned reforms struggle to produce systems-level change, highlighting the need to build stronger bridges across disciplines invested in STEM reform.

 Integrating organizational theory into the design and implementation of SIPs offers a critical pathway forward. Organizational theory provides tools to interrogate key dimensions of institutional reform, such as departmental cultures, norms, leadership structures, and power hierarchies, which shape institutional environments (NASEM, 2023; Reinholz & Apkarian, 2018). Yet, while recent scholarship has highlighted the importance of organizational change for STEM equity more broadly, there is limited research that offers concrete guidance on how to design SIPs that intentionally target institutional structures. As a result, how SIPs can function as mechanisms for systemic change remains underexplored.

## Conceptual Framework: Four Frames for Systemic Change

 To understand how STEM intervention programs can be designed to function as a mechanism for organizational change, this study draws on the four frames for systemic change (Reinholz & Apkarian, 2018). This framework conceptualizes how to create culture change within STEM departments through four interrelated dimensions: structures, symbols, power, and people. Accordingly, Reinholz and Apkarian define culture as “a historical and evolving set of *structures* and *symbols* and the resulting *power* relationships between *people*” (p. 3). This definition operationalizes culture, thus providing a framework for understanding both what factors shape culture and how to change it. Together, this framework provides a conceptual guide for designing STEM programs that account for organizational dynamics that shape STEM environments and influence the sustainability of equity-centered change.

 Each of these frames offers a distinct but complementary lens for understanding how to undertake change within higher education institutions. The **structures frame** accounts for formal systems that shape people’s actions, such as policies, practices, roles, responsibilities, and incentives. Change within this frame involves altering the structures that shape behavior, for example, tenure and promotion policies that favor research over equity-centered teaching reform.

The **symbols frame** captures the underlying logics that guide behavior, including individual values, vision, myths, knowledge, and language. This frame underscores that change requires not only a change in structure, but also in our underlying ways of thinking. The **people frame** emphasizes that organizations are composed of individuals with diverse goals, needs, and identities that influence their engagement in change processes. Change is most likely to succeed when people are connected through shared purpose, collective vision, and provided with the support necessary to enact change. Lastly, the **power frame** highlights how institutional authority (e.g., formal roles, titles) and social hierarchies (e.g., race, gender, sexual orientation) shape the dynamics of organizational change. Creating equity-centered transformation requires engaging “power holders” (Reinholz & Apkarian, 2018, p. 5) who have institutional authority to legitimize and sustain reform and redistributing power by meaningfully including historically marginalized voices in the change process.

In this study, the four frames for systemic change provided a powerful theoretical structure to analyze and interpret how STEM programs can engage in organizational change processes by focusing on shaping policies (structures), organizational narratives (symbols), stakeholder engagement (people), and decision-making hierarchies (power). The methods section below details the study’s design, data sources, and analytic process, including how the four frames allowed for an organizational change approach that is often underutilized in studies that focus on STEM programs and initiatives.

## Methods

### Research Design and Case Contexts

 This work draws on a larger study that conducted a multi-site qualitative case study (Stake, 2006; Yin, 2018) of the Meyerhoff Adaptation Project (MAP). The MAP, first started in 2013, sought to replicate the nationally recognized Meyerhoff Scholars Program (MSP) from the University of Maryland, Baltimore County (UMBC) into two predominantly white and research-intensive institutions (Hrabowski et al., 2019; Sto. Domingo et al., 2019). Using the Meyerhoff model, and with support from the Howard Hughes Medical Institute (HHMI), the University of North Carolina, Chapel Hill (UNC-CH) established the Chancellor’s Science Scholars Program (CSS) and the Pennsylvania State University, University Park (PSU) created the Millennium Scholars program (MLN).

**Figure 1.**
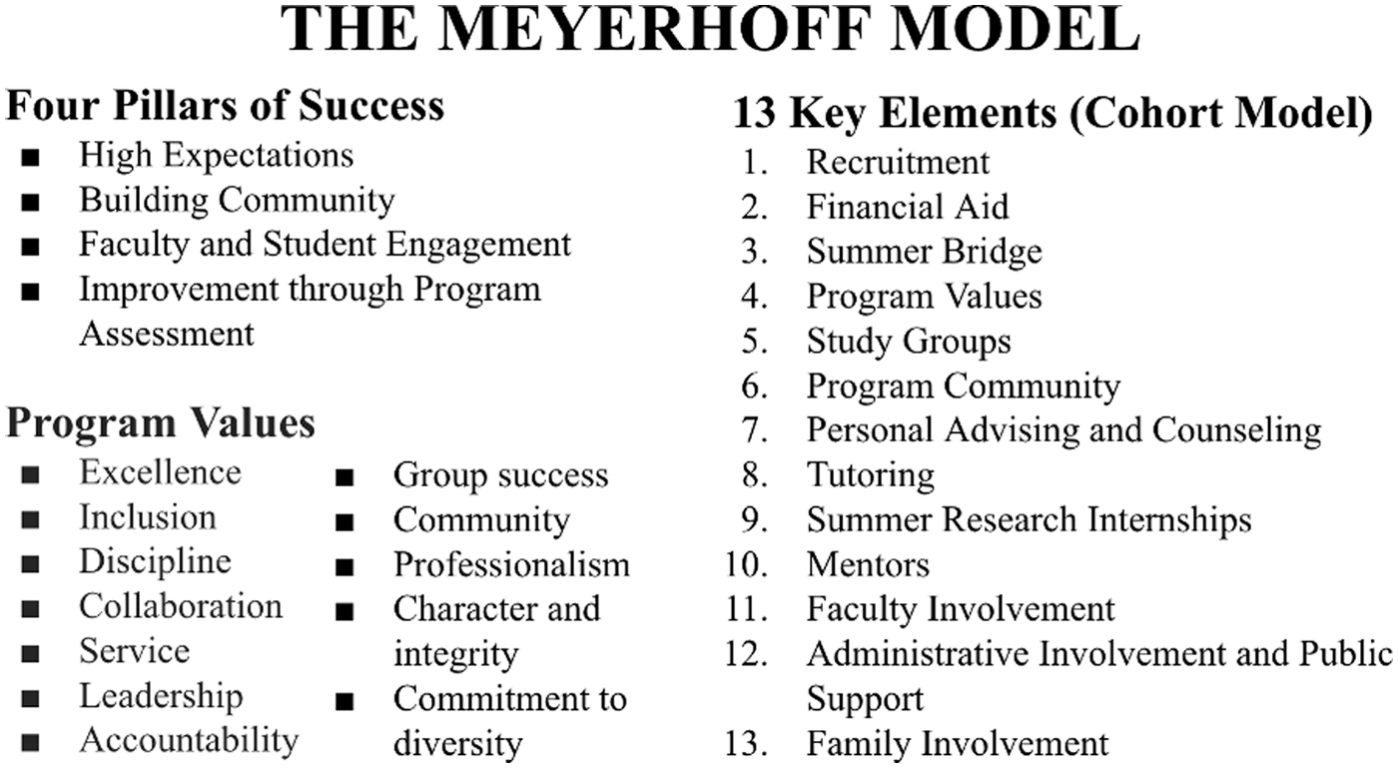
The Meyerhoff model.

The Meyerhoff program is one of the best documented STEM programs and is considered a national model for advancing underrepresented students in STEM fields. (Maton et al., 2012; NASEM, 2016; Sto. Domingo et al., 2019). Through the MAP, UNC-CH and PSU were expected to replicate all aspects of the Meyerhoff model (Figure 1) to understand whether this student-centered and culturally responsive model could be effective at institutions with drastically different racial histories, geographies, and institutional cultures. As shown in Figure 1, the model includes a wide array of programmatic components, making it resource intensive and challenging to replicate in its entirety (Hrabowski et al., 2019). While the CSS and MLN programs had aims of increasing student-level outcomes like academic performance, retention, and matriculation to STEM doctoral programs, a key underlying goal was to examine whether the development of these programs could result in inclusive institutional cultures and broader organizational change (Crimmins et al., 2017). Central to this effort are the Meyerhoff’s core values, which emphasize aspects like inclusion, collaboration, and community. Thus, replication was more than solely recreating programmatic components, it necessitated creating new cultures grounded in foundational values that seek to work toward more inclusive environments.

A multi-site qualitative case study of the MAP offered a rare and powerful opportunity to study how to design SIPs that seek to create broader change. Previous studies have found that comprehensive programs like MSP, CSS, and MLN are uncommon in higher education (Kezar & Holcombe, 2020; Kezar & Kitchen, 2020), which means limited knowledge exists regarding how they are implemented and sustained. This study is unique in that it examined the implementation of two programs (CSS and MLN) from the ground up, which drew from a proven national model. Data collected allowed for an understanding of both how programs were designed in the early years and the adjustments that were made through a 10-year period in order to enable sustainability and work toward long-term equity-centered change. In this way, the MAP served as an ideal context for investigating how to intentionally design SIPs that can function as levers for institutional transformation.

### Data Sources and Analysis

 Data for this study were drawn from a comprehensive dataset collected between 2021 and 2022 as part of an HHMI-commissioned evaluation of the MAP, which sought to examine whether the CSS and MLN programs had led to broader institutional change on their respective campuses. This data was collected nearly a decade after the initial launch of the CSS and MLN programs in 2013, offering a unique opportunity to examine how the programs have evolved over time. Data collection was undertaken by a larger research team composed of the principal investigator, a postdoctoral scholar, and several graduate students.

Data collected included 91 semi-structured interviews with 72 participants who were current and previously employed institutional administrators, program staff, faculty, and evaluators at the 3 campuses. Administrators included university presidents, provosts and vice provosts, deans of STEM colleges, and associate deans of diversity initiatives. Program staff included individuals tasked with running the day-to-day operations of the programs, including executive directors, assistant directors, and program coordinators. Faculty primarily consisted of those who had close interaction with the program scholars, such as teaching in summer bridge courses or engaging in research and mentorship with scholars. Lastly, all programs had evaluation teams led by faculty members, often from social science departments. The demographic information of participants is intentionally not reported to protect their anonymity.

This was important as some participants held their positions from program inception to the time of data collection and worked in teams of only one or two people. Moreover, programs had singular positions, such as one executive director, meaning that even assigning pseudonyms could reveal participants’ identities.

 Beyond semi-structured interviews, the larger research team conducted site visits to UMBC, UNC-CH, and PSU, in which observations were conducted along with focus groups with program scholars. The research team also engaged in document collection, including over 130 documents from student newspapers, news articles, internal program and MAP documents, and publicly available information web scraped from CSS and MLN program websites. Document collection allowed for the creation of institutional narrative reports (Yin, 2018), developed by multiple members of the broader research team, which captured unique case contexts such as university missions and histories, student body demographic information (i.e., enrollment data), and CSS and MLN program information. This extensive dataset allowed for in-depth comparisons across institutional contexts and over time.

 The author of this study has engaged in a sustained, iterative process of analysis across multiple projects, drawing on this same dataset. This began with the author’s dissertation research, which examined the life cycle of the CSS and MLN to understand how SIPs are implemented, adapted, sustained, and work toward becoming institutionalized. Subsequently, the author and the broader research team have produced manuscripts on topics like the cross-institutional mentoring relationship between HHMI, MSP, MLN, and CSS, as well as examined the institutional impact and organizational change that the CSS and MLN have had on their campuses. Overall, the author has engaged with this dataset over a 4-year period, beginning with data collection in 2021, followed by dissertation analysis, and sustained through producing manuscripts on related topics.

 For this current study, the analysis approach entailed revisiting the dataset with a specific focus on how programmatic design can function as a mechanism for organizational change, a topic that the broader research team had yet to explore. The data was re-examined through the lens of the four frames for systemic change (Reinholz & Apkarian, 2018) to identify how the programs can be designed in ways that leverage structures, symbols, people, and power to work toward being equipped to be levers of change. The author employed a two-phase cross-case analysis (Yin, 2018) to generate an in-depth and contextual understanding of the research topic and question.

The first phase entailed within-case analysis, where I examined CSS and MLN separately to understand how the local context shaped program design. This phase leveraged multiple sources of evidence (e.g., interviews, internal documents, program and university websites) to construct both narrative and visual explanations of key programmatic design dimensions such as staffing structures, reporting lines, institutional placement, and funding models. The research team used these data sources to develop chronological sequences (Yin, 2018) that traced how programs evolved over time, including major shifts in aspects like funding models and administrative placement. This within-case phase provided the necessary foundation to then compare how the organizational contexts at each institution shaped program design decisions.

In the second phase, comparative case analysis, I focused on identifying cross-site patterns but remained aware of how contextual nuances produced divergent outcomes. Comparative analysis was supported by the development of cross-site matrices (Yin, 2018), which allowed for centralized comparisons of design features and program development. The four frames for systemic change (Reinholz & Apkarian, 2018) served as an organizing structure for the cross-site comparison, enabling a systematic approach to understanding how organizational structures, symbols, power, and people play a role in program design and organizational change potential. Through cross-case analysis, the author synthesized four design principles that reflect how STEM intervention programs can be intentionally structured to create the organizational conditions necessary for equity-centered transformation.

## Findings

### Design Principles for STEM Programs That Catalyze Equity-Centered Change

 Drawing from cross-case analyses of the MAP, four interrelated principles emerged that illustrate how STEM intervention programs can be intentionally designed to advance equity-centered change. Each principle engages organizational mechanisms, including structures, symbols, people, and power, in order to work toward program sustainability and long-term success. While these principles are grounded in findings from the MAP programs, they can be applied to diverse institutional contexts as they speak to organizational processes and dynamics present across higher education institutions.

#### Principle 1: Design Programs to Disrupt Exclusionary Norms and Structures

The first principle centers on programmatic design as a form of disruption, that is, how programs can intentionally work toward challenging existing harmful structures and logics. Critiques of STEM intervention programs have pointed out that many focus narrowly on improving individual student outcomes without challenging the exclusionary cultures that pervade STEM fields and institutions. Rather than layering support onto inequitable systems, programs should aim to transform them. This is achieved by disrupting dominant norms and cultures that have excluded students from minoritized backgrounds. Findings from the MAP case study illustrate how program design can intentionally challenge such norms.

Both the CSS and MLN programs restructured the academic and social environment of STEM by fostering collaboration, community, and cultural affirmation. For example, peer support was not a byproduct of the programs, it was deliberately built into their design. From the outset, the CSS and MLN programs drew from the Meyerhoff model’s emphasis on cohort-based learning, study groups, and communal accountability. Program leaders intentionally cultivated a sense of collective identity among scholars by holding joint events, embedding peer mentoring structures, and rewarding collaboration over competition. This cultivated a culture in which collective achievement superseded entrenched STEM values of hyper-competitiveness and individualism. Too often, cultures are assumed to be fixed; however, intentional design decisions can create new norms that reflect inclusion, solidarity, and mutual success.

The CSS and MLN programs also sought to challenge deeply embedded racial bias and stereotypes about who belongs and who can succeed in STEM. At both institutions, program leaders described having to dismantle pervasive beliefs within their predominantly white institutions that framed diversity and excellence as mutually exclusive. Drawing from the Meyerhoff model, the CSS and MLN recruited high-achieving scholars, many of whom came from the top of their high school classes. Despite this, some faculty perceived these programs as remedial simply because of their high concentration of scholars of color. In response, programs worked to shift campus mindsets about who could be a scientist and succeed in STEM through public recognition of scholars, strategic storytelling, and increased institutional visibility. These strategies were especially significant at institutions like UNC-CH, which have racial histories marked by legacies of slavery and confederate monuments on campus. In this context, CSS was a counter-narrative, symbolically and materially challenging longstanding racial exclusion.

Designing for disruption challenges dominant ideologies and structures, rather than supporting students to navigate them. Moreover, it has the potential to shift norms around collaboration, merit, and racial equity by embedding values that affirm student strengths and confront the structural barriers that persist in STEM. Programs that aspire to institutional transformation cannot be neutral they must be purposefully disruptive.

#### Principle 2: Adapt Proven Models to Local Institutional Contexts

 Adapting proven models, such as the Meyerhoff program, is a common strategy institutions use to advance diversity in STEM. George et al. (2019) observe, replicating programs allow institutions to meet external pressure such as federal mandates for increased representation without having to build a program from scratch. While adapting existing models can be a productive starting point, it must be approached with a critical understanding of how local institutional context and culture will shape implementation and outcomes. Without intentional adaptation, institutions risk reinforcing status quo dynamics rather than transforming them.

 An underexplored area of research is examining how adapting models into Minority-Serving Institutions (MSIs) might differ from PWIs. As evident by the MAP data, the programs had to engage in explicit work to reframe diversity as central to excellence. However, such barriers may not be necessarily present in institutions like Hispanic-Serving Institutions (HSIs), where diversity is the norm. Research by A.-M. Núñez (2025) demonstrates that broad access HSIs approach STEM equity work through “opportunity-centered” logics that center on inclusivity, talent development, and cultural responsiveness, which directly challenge traditional STEM norms of exclusion. STEM programs enacted in these settings can leverage logics of cultural responsiveness to design programs that draw on the cultural wealth of students rather than operate in race-neutral approaches more common in PWIs. For example, programs can cultivate Latinx students’ STEM identity development through a community-centered model (Herrera & Kovats Sánchez, 2022). Indeed, HSIs could provide the necessary organizational conditions to design programs that truly serve Latinx students (Garcia et al., 2019; A.-M. Núñez, 2025), not just help them conform to exclusionary STEM cultures.

 While institutions often turn to replication to jumpstart equity efforts, true transformation requires moving beyond surface-level adoption toward contextually grounded redesign. When adapted critically, proven models can catalyze organizational learning, shift institutional logics, and embed equity into the fabric of STEM education. But this is only possible when adaptation is accompanied by intentional reflection, cultural responsiveness, and a willingness to depart from the original model when local needs demand it.

#### Principle 3: Cultivate Shared Leadership Through Intentional Collaboration

 STEM programs that aspire to create institutional change cannot operate in isolation. Instead, they must become embedded within collaborative structures that enable them to achieve shared equity leadership (Kezar et al., 2021), an approach where the responsibility of advancing equity is distributed to members across the institution.

This principle is especially important within PWIs, where faculty and leaders of color often carry a disproportionate burden for equity work, a dynamic described as the minority tax (Rodríguez et al., 2015). Findings from the MAP show that the CSS and MLN programs sought to shift this dynamic by intentionally involving institutional leaders across racial lines. Though many of the program staff and leaders were people of color, they also secured buy-in from faculty and central administrators who were often white. As one top administrator explained, “you don’t have to be a minority to help minority students.” Through this lens, equity becomes a collective responsibility, not an individual obligation. Thus, collaboration is not simply about improved coordination it is about transforming how institutions operate and shifting expectations about who is responsible for equity work.

Intentional collaboration was central to the design and sustainability of the MAP programs. Both UNC-CH and PSU developed steering committees and advisory boards composed of cross-rank, cross-role, and cross-unit participants. These structures distributed leadership and enabled collective ownership of the programs’ success. They also facilitated relational trust, alignment across silos, and campus-wide buy-in. As Kezar and Holcombe (2017, 2020) argue, integrated programs are more effective when collaborative leadership ensures that program components are mutually reinforcing and informed by diverse perspectives.

 STEM programs that strive to create change must activate institutional actors toward collective transformation. Findings from the MAP reinforce that when equity work is relegated to a few committed individuals, often people of color, it risks isolation and burnout. STEM programs can build intentional collaborative structures that distribute labor, cultivate shared ownership, and integrate equity into institutional norms. This shift allows programs to move from being isolated projects to intentional efforts capable of organizational change.

#### Principle 4: Sustain Programs Through Structural Commitments

 A persistent challenge across STEM equity efforts is ensuring the sustainability of programs beyond initial grant funding (Rincón & George-Jackson, 2016). While many STEM intervention programs are launched with external or temporary funds, they often struggle to endure without long-term commitments from core institutional funds (Cobian & Ramos, 2021; Gomez et al., 2021; Rincón & George-Jackson, 2016). When institutions fail to integrate programs into internal budgets, it can lead to staffing instability, diminished service delivery, and constant pressure on program administrators to secure outside funding (Cobian & Ramos, 2021; Gomez et al., 2021; Rincón & George-Jackson, 2016). These fragile funding structures make programs particularly vulnerable to shifting institutional priorities and macroeconomic changes.

 The CSS and MLN programs were initiated through external support from HHMI, however, they were required to transition to institutional support from UNC-CH and PSU after 5 years (Hrabowski et al., 2019). Both campuses underwent multiple iterations of program placement and funding model development to ensure long-term sustainability. Strategies included placing the programs into high-level units like the Office of the Provost for MLN and Honors Carolina for CSS, allowing them to secure institutional visibility and legitimacy. Additionally, both programs were framed as signature programs and incorporated into university-wide fundraising campaigns, which helped stabilize their financial foundations. Notably, at the time of data collection (2021–2022), both institutions faced significant financial pressures due to the COVID-19 pandemic. These constraints led to adjustments such as reducing the number of scholars admitted that year.

Insights from the MAP demonstrate that STEM programs require deep institutional commitments that embed them into the core structure of the institution, rather than operating on the margins. Ray’s (2019) concept of racialized decoupling explains how equity efforts are often decoupled from formal policies and practices allowing them to be visible markers of diversity but lacking the ability to change power structures that enable exclusion. To avoid this fate, STEM programs must be structurally integrated into institutional design through strategic program placement, sustainable funding models, and aligned with institutional missions. Only through this level of commitment can they function as levers of transformation, rather than temporary add-ons fighting for survival.

## Discussion, Implications, and Future Directions

 This study examined how STEM programs can be intentionally designed to serve as mechanisms for equity-centered organizational change. Drawing on the four frames for systemic change (Reinholz & Apkarian, 2018), the findings underscore that institutions seeking to enact change must leverage their organizational structures, logics, people, and power toward transformation. Programs that reconfigure exclusionary cultural norms, adapt to their local context, redistribute leadership, and are sustained through structural commitments are better positioned to disrupt entrenched inequities within STEM fields and higher education institutions. As STEM intervention programs continue to be a dominant strategy for addressing disparate outcomes, future efforts must focus on designing them as organizational catalysts rather than individual-level remedies.

### Implications for Practice

 Institutional stakeholders who seek to employ STEM intervention programs to create equity-centered change can leverage the principles outlined in this study to make intentional organizational design choices. In practice, this could include prioritizing collaborative leadership structures, such as advisory boards, cross-unit committees, that distribute responsibility for change and build relational trust across campus. Moreover, stakeholders become aware of the importance of integrating programs into institutional infrastructures through strategic plan inclusion, placement within high-visibility administrative units, and funded through stable models that allow long-term stability. Through these practices, campus leaders and stakeholders can ensure that STEM programs become part of the institution’s core rather than peripheral initiatives dependent on individual champions or temporary funding grants.

### Implications and Future Directions for Research

 Future research should continue to explore how programs designed to support underrepresented students can catalyze organizational change in durable ways. Researchers might further explore how programs can influence institutional cultures and logics, resource distribution, and norms of shared equity leadership. Importantly, research must be conducted in different institutional types, like Hispanic-Serving Institutions to understand how they might differently leverage institutional assets, like opportunity-centered logics (A.M. Núñez, 2025), and understand unique barriers they might encounter. Research conducted at HSIs has shown that culturally responsive and community-rooted practices (Herrera & Kovats Sánchez, 2022) are critical for Latinx student success. Building on this, researchers should investigate how STEM programs can be designed to affirm students’ cultural wealth and engage in servingness (Garcia et al., 2019), particularly at the departmental level where STEM cultures are most pervasive. In studying STEM initiatives, we should center frameworks like servingness, which center power and race as key areas to be addressed to achieve sustained organizational change.

### Implications for Policy

The 2025 wave of federal funding cuts has placed STEM equity initiatives at risk nationwide, threatening programs that have long expanded opportunity for underrepresented students (Mervis, 2025). These losses can lead to dire consequences for educational access, workforce diversity, and the nation’s scientific competitiveness. Policymakers must prioritize restoring and expanding federal investments for equity-centered STEM initiatives. Institutions cannot carry the responsibility of equity alone, especially institutions like HSIs, which have historically and currently face underfunding (A. M. Núñez et al., 2015). Divestment from STEM efforts does not just threaten programs, it threatens our collective progress. Without these critical investments, we risk losing an entire generation of diverse scientists whose contributions are vital to the nation’s future.
